# Supplementary figures and images for: Formulation and Functional Characterization of a Cannabidiol-Loaded Nanoemulsion in Canine Mammary Carcinoma Cells
Source: Pharmaceutics. 2025 Jul 26;17(8):970. doi: 10.3390/pharmaceutics17080970 (PMC12389271; doi:10.3390/pharmaceutics17080970)

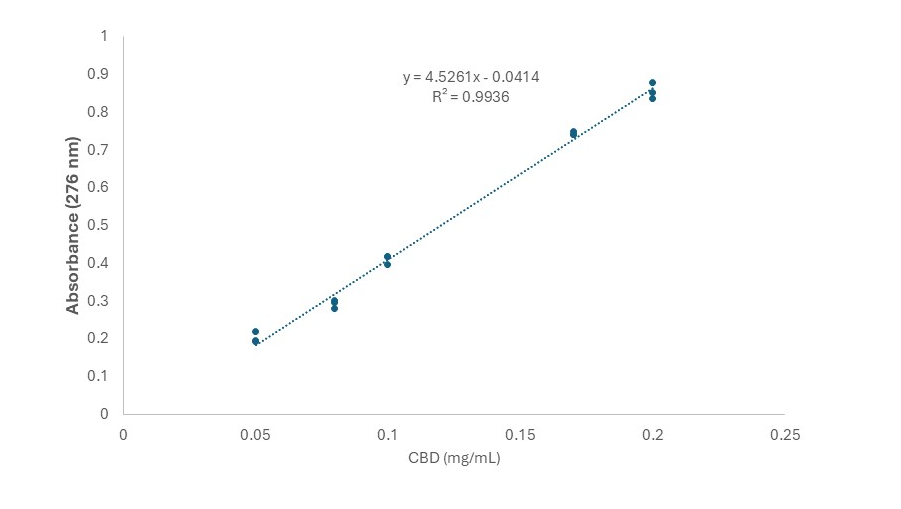

Supplement: Supplementary file 1 [file pharmaceutics-17-00970-s001.zip › Figure S1.tiff]

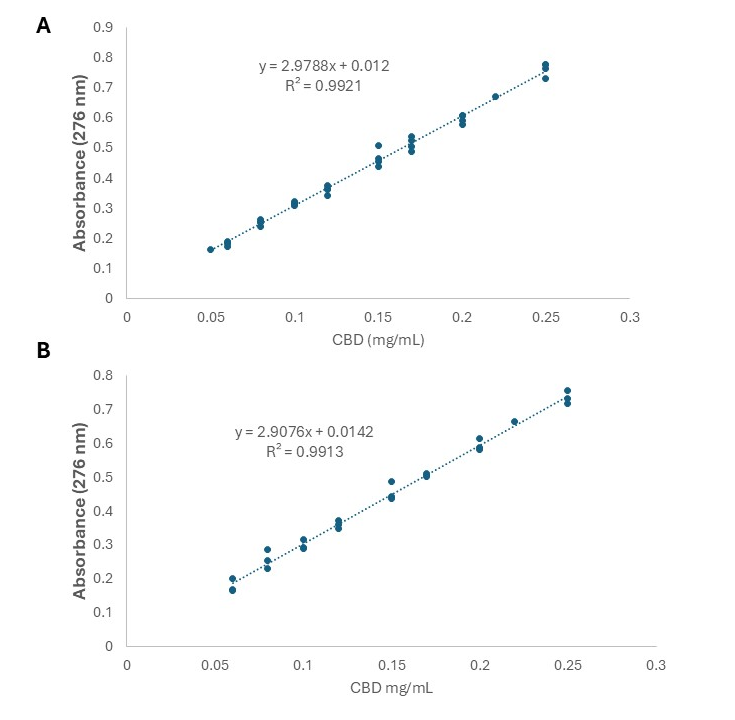

Supplement: Supplementary file 1 [file pharmaceutics-17-00970-s001.zip › Figure S2.tiff]
